# Tunable Two-Layer Dual-Band Metamaterial with Negative Modulus

**DOI:** 10.3390/ma12193229

**Published:** 2019-10-02

**Authors:** Limei Hao, Meiling Men, Yazhe Wang, Jiayu Ji, Xiaole Yan, You Xie, Pengli Zhang, Zhi Chen

**Affiliations:** 1 Department of Applied Physics, Xi’an University of Science and Technology, Xi’an 710054, China; sophismile_mml@163.com (M.M.); marpessa_wyz@163.com (Y.W.); jiayuji19980125@sina.com (J.J.); yanxl@xust.edu.cn (X.Y.); xieyou@xust.edu.cn (Y.X.); zhangpl1983@163.com (P.Z.); 2 Department of Applied Physics, Northwestern Polytechnical University, Xi’an 710129, China

**Keywords:** acoustic metamaterial, two-layer SHSs, dual-band, two spring oscillators in series, two negative moduli

## Abstract

A tunable dual-band acoustic metamaterial (AM) with nested two-layer split hollow spheres (TLSHSs) is presented here, which was achieved by adjusting the hole diameter and the ratio of the two layers’ volumes. This work comprises theoretical and numerical studies. Based on sound-force analogy (SFA), TLSHSs can be considered equivalent to a model of two spring oscillators in series. The equations of two resonant frequencies were derived, which precisely provided the relation between two resonant frequencies and the hole diameter as well as the ratio of the two layers’ volumes. The analytical formulas and simulation results by the finite element method (FEM) showed that there were two resonant frequencies for the TLSHSs, and their dynamic modulus became negative near the resonant frequencies. As the the diameter of two holes increased, both of the resonant frequencies underwent a blue shift. As the relative volume ratio increased, both of the resonant frequencies underwent a red shift. The calculation and simulation results were in good agreement. This kind of precisely controllable dual-band AM with negative modulus can easily be coupled to other structures with negative mass density, thereby achieving a double-negative AM in an expected frequency range.

## 1. Introduction

Acoustic metamaterial (AM) is an artificial material that exhibits excellent physical properties, including negative effective mass density [1] and negative effective modulus [2]. Therefore, AMs have attracted considerable research attention in the past two decades. Through the design of various novel structures, AMs can be used in many different areas, such as super-resolution [3], sound cloaking [4], subwavelength imaging [5], superlensing [6], negative index [7], reversed Doppler shifts [8], sound absorption [9], and so on. Thus, the design of the unit structure is of great importance in the application of AMs.

The use of local resonance units is a feasible way to design acoustic metamaterials. Liu et al. [1] fabricated the first AM with local resonant units and it can obtain negative effective density. After that, local resonance AMs have been studied widely [10,11,12,13,14]. In 2006, Fang et al. [2] designed a one-dimensional array consisting of a split hollow cavity of subwavelength, and it achieved negative effective modulus at the ultrasonic frequency. In 2015, Jing et al. [15] proposed a balloon-like soft resonator based on monopolar resonance, and it realized negative effective modulus. However, the disadvantage of local resonance is that different structural units have only a single resonant frequency.

At present, AMs with multiple frequencies can be prepared by adding heterogeneous structural units. Fan el at. [16] designed an AM using periodical multilayer perforated plates coated with a membrane, and multiple frequencies were obtained. A broad frequency range from 50 to 1000 Hz was realized by membrane reflectors with multiple mass blocks [17]. A local resonant acoustic metamaterial including eight units of periodic coaxial multicoated inclusions achieved two broadbands [18]. A broadband AM was realized by an ultrathin multisplit unit [19]. Shen et al. [20] proposed a unit of double or quadruple branch openings in a 1-D waveguide, and a broadband was realized by periodically arranging these units. A multiband AM with negative modulus was achieved by composing seven split hollow spheres with different sizes [21].

In short, multiple frequencies or broadband can be obtained by assembling multiple heterogeneous units, which makes the structure bulky and complex. Our research group [22] has presented a nested structural unit, and it can effectively reduce the size. Furthermore, our numerical results demonstrate that the number of multiband resonant frequencies is regulated by adjusting the number of multilayer resonators. Because of the special response of the local resonance, it has become an urgent task to accurately predict the resonant frequencies of structural units with the different structural parameters. However, little attention has been paid to the effect of the structural parameters of a nested multilayer unit on the transmission properties.

Here, the influence of the structural parameters of two-layer split hollow spheres (TLSHSs) on the resonant frequency was examined from the perspective of sound-force analogy (SFA) theory and a simulated method. Furthermore, the effects of the hole diameter, the relative diameter of two holes, and the relative volume ratio on the transmission properties were analyzed in detail with a simulation method.

## 2. Model and Simulation

**Figure 1:**
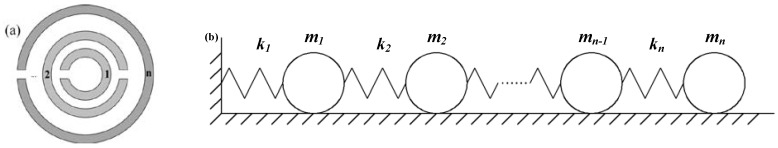
(**a**) The cross section of multilayer split hollow spheres (MLSHSs). (**b**) Schematic diagram of multiple spring oscillators in series.

Multilayer SHSs (MLSHSs) are nested by multiple SHSs of different sizes, the geometric illustration of which is shown in Figure 1a. Notice that the sound wave enters the multilayer SHSs from the outermost hole, then crosses the interlayer between the interlayers and goes into the inner cavity through the inner hole, and finally gets into the innermost cavity. Thus, these holes are equivalent to masses ($m_{1}$, $m_{2}$… and $m_{n}$). The innermost cavity and the interlayer can store sound energy, so that they can be considered equivalent to springs ($k_{1}$, $k_{2}$ … and $k_{n}$). According to the principle of the sound-force analogy, multilayer SHSs can be equivalent to multiple spring oscillators in series, as shown in Figure 1b. By the force analysis of $m_{1}$, $m_{2}$ … and $m_{n}$, we have Equations (1)–(4):

(1) $$m_{1}\frac{d^{2}x_{1}}{dt^{2}}+k_{1}x_{1}-k_{2}(x_{2}-x_{1})=0$$

(2) $$m_{2}\frac{d^{2}x_{2}}{dt^{2}}+k_{2}(x_{2}-x_{1})-k_{3}(x_{3}-x_{2})=0$$

(3) $$m_{i}\frac{d^{2}x_{i}}{dt^{2}}+k_{i-1}(x_{i-1}-x_{i-2})-k_{i}(x_{i}-x_{i-1})=0$$

(4) $$m_{n}\frac{d^{2}x_{n}}{dt^{2}}+k_{n-1}(x_{n-1}-x_{n-2})-k_{n}(x_{n}-x_{n-1})=0.$$

Let $x_{1}=A\cos(\omega t)$, $x_{2}=B\cos(\omega t)$, ... $x_{i}=I\cos(\omega t)$, and ... $x_{n}=N\cos(\omega t)$. Substituting them into the above equations, we can obtain the characteristic equation of the angular frequency $\omega_{1}$, $\omega_{2}$, ..., $\omega_{n}$. Solving this equation, we can get the expression of $\omega_{1}$, $\omega_{2}$, ..., $\omega_{n}$, and then obtain $f_{1}$, $f_{2}$, ..., $f_{n}$ by $f_{i}=\frac{\omega }{2\pi }$.

**Figure 2:**
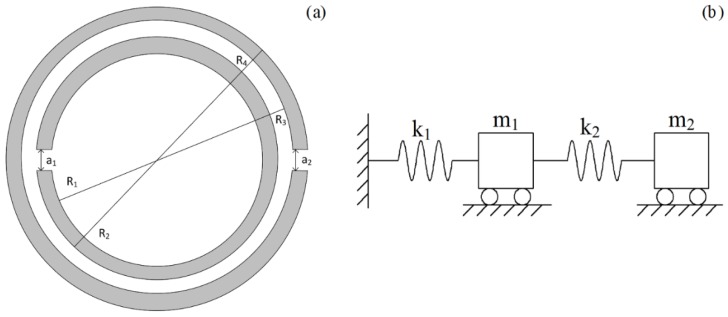
(**a**) The cross section of two-layer split hollow spheres (TLSHSs). (**b**) Schematic diagram of spring oscillators in series.

Similarly [23], TLSHSs are nested by two SHSs of different sizes, the schematic of which is shown in Figure 2a. $R_{1}$, $R_{2}$, and $a_{1}$ represent the inner radius, outer radius, and the split hole diameter of the inner layer sphere, respectively. $R_{3}$, $R_{4}$, and $a_{2}$ represent that of the outer layer sphere, respectively. TLSHSs can be considered equivalent to two spring oscillators in series, as is shown in Figure 2b. When the spring oscillator in series is in steady state, the oscillator function is composed of two resonant frequency components. Based on the above analysis, the formula of the resonant frequency of the TLSHSs is obtained by taking n = 2, and then the two resonant frequencies satisfy Equations (5) and (6) as follows [24,25]:

(5) $$f_{1}=\frac{\sqrt{\frac{k_{2}}{2m_{1}m_{2}}(\alpha -\sqrt{\alpha^{2}-4\beta })}}{2\pi }$$

(6) $$f_{2}=\frac{\sqrt{\frac{k_{2}}{2m_{1}m_{2}}(\alpha +\sqrt{\alpha^{2}-4\beta })}}{2\pi }$$

where the effective sound mass of the inner split hole is expressed as $m_{1}=\frac{\rho l_{1}}{\pi {\left(\frac{a_{1}}{2}\right)}^{2}}$, and that of the outer hole as $m_{2}=\frac{\rho l_{2}}{\pi {\left(\frac{a_{2}}{2}\right)}^{2}}$. The elastic coefficient of the inner cavity is expressed as $k_{1}=\frac{\rho c^{2}}{\frac{4}{3}\pi R_{1}^{3}}$, and that of the interlayer as $k_{2}=\frac{\rho c^{2}}{\frac{4}{3}\pi (R_{3}^{3}-R_{2}^{3})}$. In the formula, $\alpha =m_{1}+m_{2}+\frac{k_{1}}{k_{2}}m_{2}$, and $\beta =\frac{k_{1}}{k_{2}}m_{1}m_{2}$. The mass density and velocity of sound in the air medium are denoted by ρ and c, respectively. The effective length of the inner hole and outer hole is denoted by $l_{1}$ and $l_{2}$, respectively.

COMSOL Multiphysics with the finite element method (FEM) was used to establish and simulate the TLSHSs, where the Thermal Acoustic Module or the Pressure Acoustic Module was selected to estimate the effect of the thermoviscous losses on the acoustic properties, separately [26]. TLSHSs were placed in the middle of the waveguide. The left side of the waveguide was set to an incident boundary, the right side was set to a matched boundary, and the other side of the waveguide was set to a hard boundary. Sound went inside from the left of the waveguide and perpendicularly entered the outer hole of the TLSHSs. Further, the inner part of the waveguide and the cavity in the TLSHSs were provided with air medium. Considering the thermoviscous loss, the air was set as a viscous flow. The Thermal Acoustic Module was applied to the narrow domains of the interlayer and the two holes, and the Pressure Acoustic Module was applied to the inner cavity and the other domain. In the calculation, the density of the TLSHSs was 1000 kg/m^3^, the Young’s modulus was set to 2 × 10^8^ Pa, and the Poisson’s ratio was 0.37. The density of air was 1.29 kg/m^3^, the speed of sound in air was 320 m/s, and the attenuation coefficient was 0.01 m^−1^. Furthermore, we primarily set $R_{1}=4$ mm, $R_{2}=4.5$ mm, $R_{3}=5.37$ mm, $R_{4}=5.87$ mm, $a_{1}=0.6$ mm, and $a_{2}=0.6$ mm.

From Equations (5) and (6), the values of $f_{1}$ and $f_{2}$ are dependent on the diameter of the holes, the volume of the cavity, and the interlayer volume of the two layers. Therefore, the effects of the diameter of the two holes and the relative volume of the two layers on the transmission properties of the TLSHSs were studied in detail.

## 3. Results and Discussion

### 3.1. Effect of the Same Hole Diameters of TLSHSs on Transmission Properties

Based on the analysis in Section 2, the diameters of the inner and outer holes were set to be the same, and they are denoted by *a*. The effect of the hole diameter of TLSHSs on the transmission properties was analyzed. Here, the hole diameter a was set to 0.2, 0.4, 0.6, 0.8, and 1 mm, respectively, and the other parameters are listed in Section 2.

**Figure 3:**
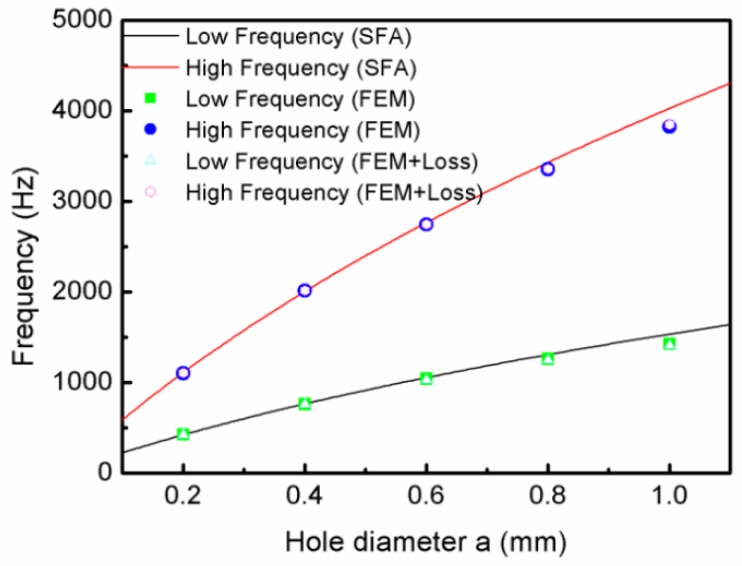
The relationship curves of the hole diameter and the resonant frequencies.

Figure 3 shows the relationship curves of the hole diameter and the resonant frequencies, where the solid square and circle symbols are the low- and high-frequency values, respectively, of the simulation without loss; the hollow triangle and circle symbols are the low- and high-frequency values, respectively, of the simulation with loss; and the lines are the calculation curves resulting from Equations (5) and (6). It is shown in Figure 3 that there were two resonant frequencies for every TLSHS. It is worth noting that the simulation results were in good agreement with the theoretical results. In addition, the simulated low- and high-frequency values without and with loss were basically the same. This is, perhaps, because the thermoviscous loss contributions can be neglected due to the relatively large interlayer cavity [27]. Furthermore, with the increase of the hole diameter, the difference between the low- and high-frequency values became larger. This was because, as the diameter increased, all of the $m_{1}$, $m_{2}$, α, and β reduced; $f_{1}$ increased slowly; and $f_{2}$ increased quickly, which made the difference larger.

**Figure 4:**
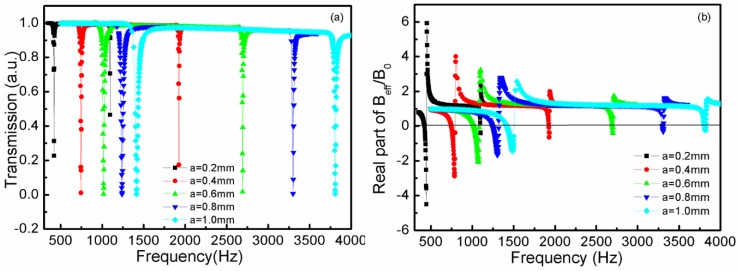
The curves of the TLSHSs with various hole diameters: (**a**) transmission and (**b**) effective modulus.

Figure 4a shows the transmission curves of the TLSHSs with various hole diameters. As seen in Figure 4a, as the two hole diameters increased, the two resonant frequencies underwent a blue shift. For example, when the diameter was 0.2 mm, the low-frequency value was 420 Hz and the high-frequency value was 1096 Hz, whereas when the diameter was 1.0 mm, the low-frequency value was 1414 Hz and the high-frequency value was 3808 Hz. The reason for this is that when the hole diameter grew, all of the $m_{1}$, $m_{2}$, α, and β decreased, and then $f_{1}$ and $f_{2}$ increased according to Equations (5) and (6).

Figure 4b shows the effective modulus curves of the TLSHSs with various hole diameters [28]. As shown in Figure 4b, the negative moduli could be realized around the low and high resonant frequencies. In addition, as the hole diameter increased, the negative modulus value near the low resonant frequency gradually became small, while that near the high resonant frequency slowly became small. For example, near the low frequency, when the diameter was 0.2 mm, the value of the negative modulus was −4.76, whereas when the diameter was 1.0 mm, the value was −1.47. Near the high frequency, the value of the negative modulus was −0.44 when the diameter was 0.2 mm, while when the diameter was 1.0 mm, it was −0.27.

### 3.2. Effect of the Hole Diameters of TLSHSs on Transmission Properties

In order to discuss the effect of the relative diameter of the two holes on the transmission properties, TLSHSs with different inner and outer hole diameters were divided into two groups. For group 1, the diameter of the outer hole was fixed at 0.6 mm, and that of the inner hole varied from 0.2 to 1.0 mm. For group 2, the diameter of the inner hole was fixed at 0.6 mm, and that of the outer hole varied from 0.2 to 1.0 mm.

#### 3.2.1. Inner Hole Diameters

Group 1 was catalogued in order to master the influence law of the inner hole diameter on the transmission characteristics.

**Figure 5:**
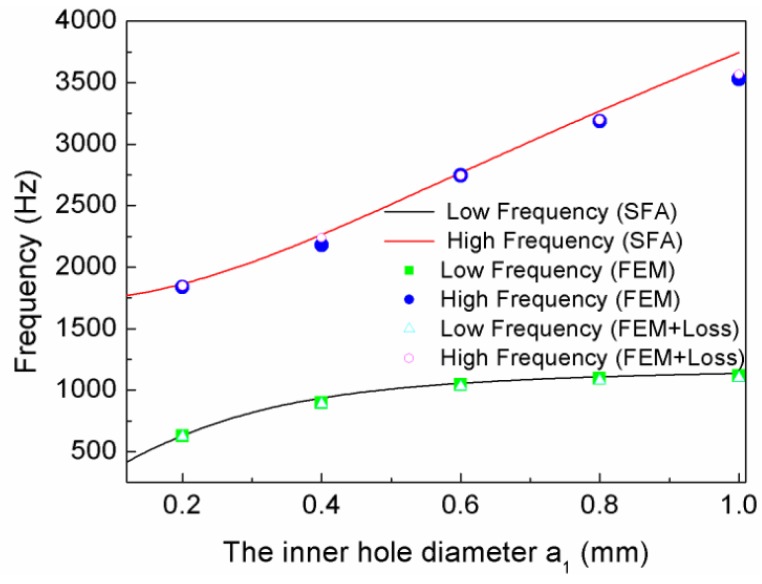
The relationship curves of the inner hole diameter and resonant frequencies.

Figure 5 shows the relationship curves of the inner hole diameter and the resonant frequencies, and the solid square and circle symbols are the low- and high-frequency values, respectively, of the simulation without loss; the hollow triangle and circle symbols are the low- and high-frequency values of the simulation with loss; and the solid curve is the calculation curves according to Equations (5) and (6). Figure 4 shows that there were two resonant frequencies for every TLSHS, and this number was independent of the diameter of the inner hole. Furthermore, the simulation and theoretical results were in good agreement. In addition, when the diameter of the inner hole was enhanced, the gap between the two frequencies became wider. The reason for this is that, as the inner hole diameter increased, $m_{1}$ reduced and both α and β decreased. Thus, according to Equations (5) and (6), $f_{1}$ increased slowly and $f_{2}$ increased quickly.

**Figure 6:**
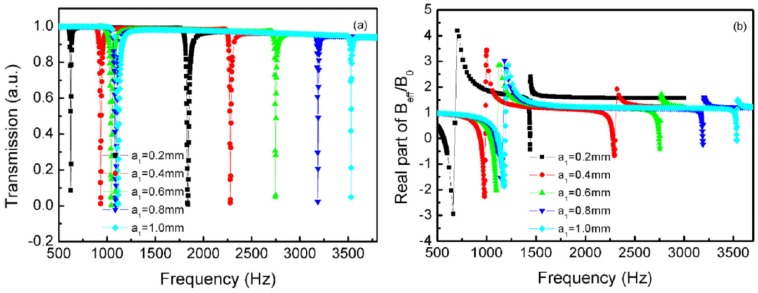
The curves of TLSHSs with various the inner hole diameters: (**a**) transmission and (**b**) effective modulus.

Figure 6a demonstrates the transmission curves of the TLSHSs with different inner hole diameters. It is shown that with the increase of inner hole diameter, the two resonant frequencies increased. For example, when the inner diameter was 0.2 mm, the low-frequency value was 624 Hz and the high-frequency value was 1838 Hz, whereas when the inner diameter was 1.0 mm, the low-frequency value was 1116 Hz and the high-frequency value was 3530 Hz. In addition, the negative moduli could be realized around the low and high resonant frequencies, as shown in Figure 6b.

#### 3.2.2. Outer Hole Diameters

Group 2 was catalogued in order to master the influence law of the outer hole diameter on the transmission characteristics.

**Figure 7:**
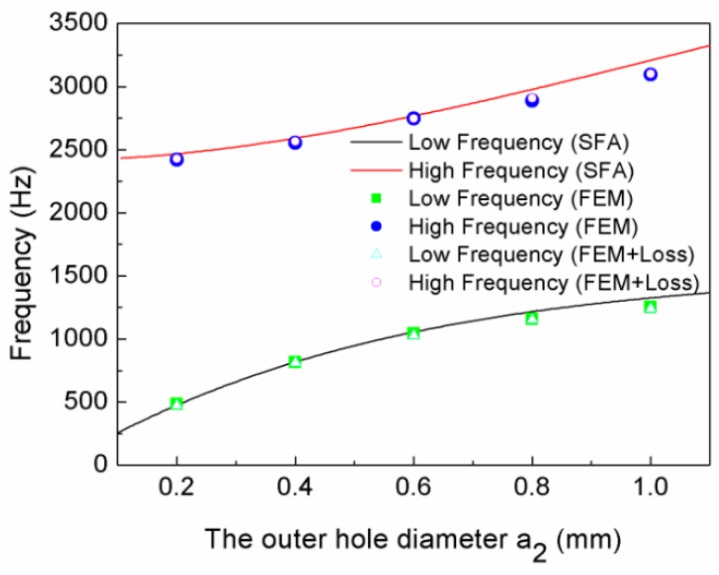
The relationship curves of the outer hole diameter and resonant frequencies.

Figure 7 depicts the relationship curves of the outer hole diameter and the resonant frequencies. There were two resonant frequencies for every TLSHS, and the simulation values were consistent with the theoretical values, as shown in Figure 7. Furthermore, the gap of the two frequencies varied slightly with the enlarging of the outer hole diameter. For instance, when the outer hole diameter was 0.2 mm, the gap was 1936 Hz, and when the diameter was 1.0 mm, the gap was 1872 Hz.

**Figure 8:**
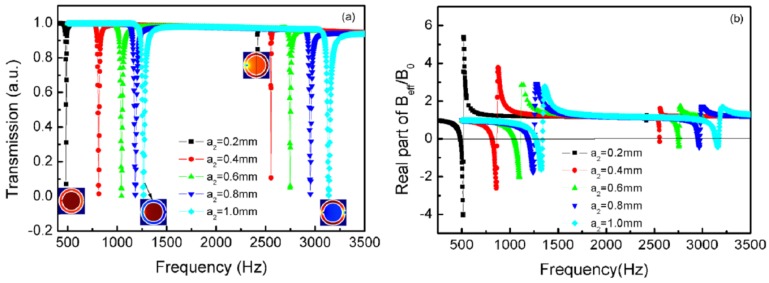
The curves of TLSHSs with various outer hole diameters: (**a**) transmission and (**b**) effective modulus.

Figure 8a plots the transmission curves of the TLSHSs with various outer hole diameters. It is shown that with the increase of the outer hole diameters, both of the resonant frequencies underwent blue shifts. For the low frequency, all of the transmittance was less than 0.1, whereas, for the high frequency, the value of the transmission dips enhanced gradually with the increase of the outer hole diameters. For example, when the diameter of the outer hole was 0.2 mm, the transmittance at the high frequency (2420 Hz) was 0.84, whereas when the diameter was 1.0 mm, the transmittance (3138 Hz) was 0.004. As shown in the inset of Figure 8a, for the high frequency, when the outer hole was 0.2 mm, the local resonance occurred near the outer hole, and then the resonance was so weak that the transmittance was high. When the inner hole was 1.0 mm, the local resonance occurred near both of the inner and outer holes, and the resonance was so strong that the transmittance was low.

The effective modulus curves of the TLSHSs with various outer hole diameters are given in Figure 8b, and the negative moduli could be realized around the low and high resonant frequencies, except that the modulus near the high resonant frequency of the TLSHSs with an outer hole diameter of 0.2 mm was positive.

In short, two transmission dips underwent a significant blueshift by increasing either of the two holes. When the outer hole was very small, there only existed one deep transmission dip at the low frequency, and the negative modulus could be realized. However, when the outer hole was big, there were two deep transmission dips, and two negative moduli could be obtained.

### 3.3. Effect of the Relative Volume of TLSHSs on Transmission Properties

In order to investigate the influence of the relative volume of TLSHSs on the transmission properties, the volume of the inner cavity was fixed at $2.67\times 10^{-7}m^{3}$*,* and the ratio of the two layers’ volumes was denoted by n varying from 0.53 to 3.94.

**Figure 9:**
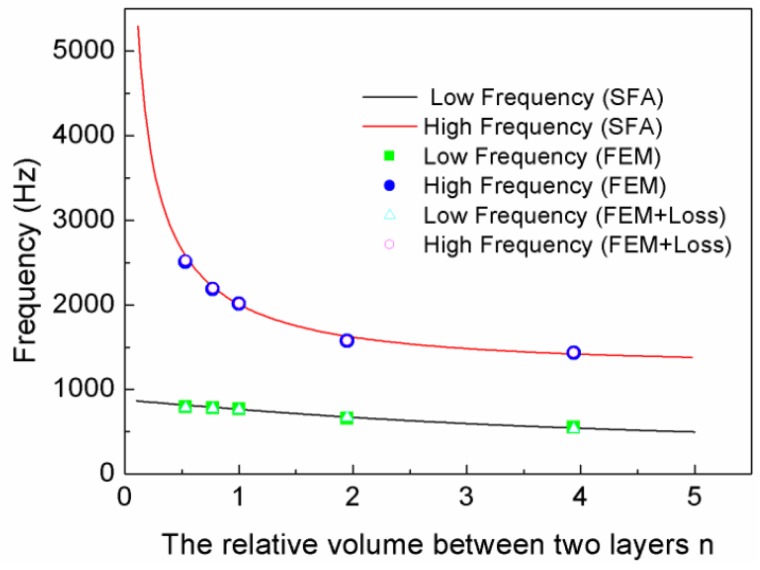
The relationship curves of the relative volume and resonant frequencies.

Figure 9 displays the relationship curves of the relative volume and the resonant frequencies. It can be seen from Figure 9 that there were two resonant frequencies for every TLSHS, and the simulation values coincided with the theoretical values. Furthermore, the gap between the two frequencies narrowed as the relative volume increased. It is thought that when the relative volume enlarges, $k_{2}$ decreases, but α and β increase. Therefore, $f_{1}$ decreased slowly, $f_{2}$ decreased quickly, and then the gap narrowed based on Equations (5) and (6).

**Figure 10:**
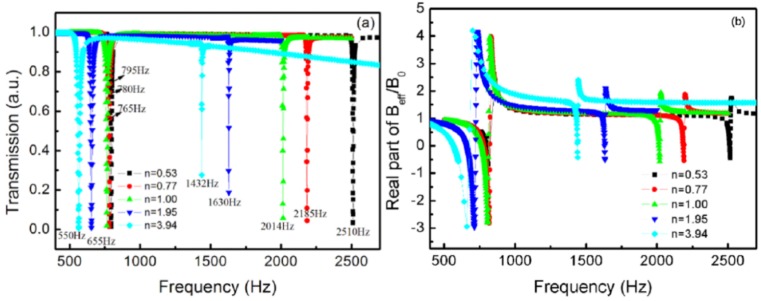
The curves of TLSHSs with various relative volumes: (**a**) transmission and (**b**) effective modulus.

Figure 10a depicts the transmission curves of the TLSHSs with different relative volumes. With the enlargement of the relative volume, the two resonant frequencies were redshifted. Furthermore, the negative moduli could be realized around the low and high resonant frequencies, as shown in Figure 10b. For the low frequency, the transmittance gradually improved with the increase of n. For example, when the relative volume was 0.53, the transmittance at the low frequency of 795 Hz was 0.014. When the relative volume was 3.94, the transmittance at the low frequency of 550 Hz was 0.0067. This was because the majority of the energy was stored in the inner cavity, and the energy between the two layers gradually enhanced (see Figure 10). The strong resonance mainly occurred near the inner hole, and that near the outer hole strengthened gradually. Thus, the transmission dip was deeper.

**Figure 11:**
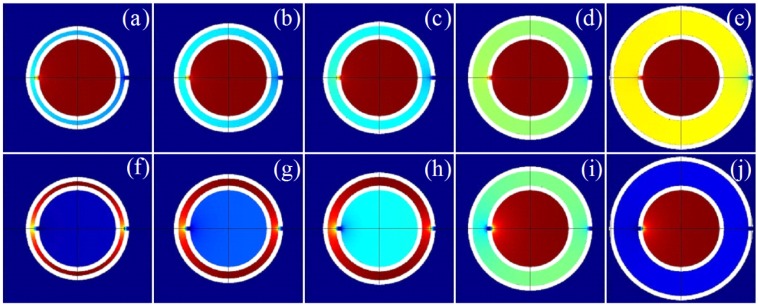
The energy distributions of TLSHSs at the resonant frequencies of (**a**) 795, (**b**) 780, (**c**) 765, (**d**) 655, (**e**) 550, (**f**) 2510, (**g**) 2185, (**h**) 2014, (**i**) 1630, and (**j**) 1432 Hz.

For the high frequency, the transmittance gradually reduced with the increase of n. For example, when the relative volume was 0.53, the transmittance at the high frequency of 2520 Hz was 0.076. When the relative volume was 3.94, the transmittance at the high frequency of 1432 Hz was 0.27. This was because when n was smaller than or equal to 1, the energy was stored between the two layers, and the local resonance occurred near both the inner and outer holes. Thus, the strong resonance resulted in low transmittance. When n was greater than 1, the majority of the energy was stored in the inner cavity, and the local resonance occurred only around the inner hole. Therefore, the transmission dip became shallower (see Figure 11).

## 4. Conclusions

In summary, we presented here a tunable two-band metamaterial with negative modulus, and its local resonance cell consisted of TLSHSs in this work. Two resonant frequencies were accurately derived by a model of two spring oscillators in series. The results showed that there were two resonant frequencies for the TLSHSs. By increasing either of the two holes, the values of the two resonant frequencies underwent a blueshift. With the enlarging of the relative volume, the two resonant frequencies underwent a redshift. Furthermore, two negative moduli could be realized around the low and high resonant frequencies. It is worth noting that the simulation results were in good agreement with the theoretical results. Thus, two resonant frequencies can be precisely modulated by the analytical formulas, and we can easily obtain a controllable unit structure with two negative moduli, which provides a basis for optimizing double-negative AMs.
